# Commentary on: Increased stiffness of omental arteries from late pregnant women at advanced maternal age

**DOI:** 10.1042/BSR20231291

**Published:** 2023-11-30

**Authors:** Anna E. Stanhewicz, Kelsey S. Schwartz, Ruda Lee

**Affiliations:** Department of Health and Human Physiology, University of Iowa, IA, U.S.A.

**Keywords:** aging, cardiovascular physiology, pregnancy

## Abstract

Worldwide, pregnancy at age 35 or older, termed ‘advanced maternal age (AMA)’, is increasing exponentially. As the incidence of pregnancy at AMA has increased, a growing body of evidence has suggested that AMA is also associated with increased risk for adverse maternal and fetal outcomes outside of genetic anomalies. Importantly, despite the mounting evidence and the increased global risk of adverse perinatal outcomes observed, few studies have examined the potential mechanisms underlying this elevated risk in pregnant people ≥35 years of age. Wooldridge and colleagues begin to address this gap in the literature. In their recent report, they examine vessel stiffness in omental resistance vessels obtained from pregnant individuals ≥35 years of age compared with pregnant individuals <35 years of age. Omental arteries were isolated and assessed via pressure myography (mechanical properties) and histological analysis for collagen and elastin content. Overall, the findings from this investigation report that maternal resistance arteries collected from women of AMA were less compliant and had less elastin than arteries obtained from women <35 years of age, suggesting that maternal resistance vessel stiffening in AMA may contribute to increased risk of adverse pregnancy outcomes. The authors should be commended for completing these studies in human resistance vessels, which now open new avenues for investigation and provoke a cascade of questions related to maternal cardiovascular adaptations to pregnancy in women ≥35 years of age.

## Commentary

Pregnancy at age 35 or older, termed ‘advanced maternal age (AMA)’, is increasing exponentially in developed countries worldwide [1–3]. Historically, this age cutoff was defined based on evidence of declining fertility and increased risk for genetic abnormalities in the offspring of pregnant individuals over the age of 35. As the incidence of pregnancy at AMA has increased, a growing body of evidence has suggested that AMA is also associated with increased risk for adverse maternal (e.g. preeclampsia, gestational diabetes, and cesarean delivery) and fetal (e.g. small for gestational age, pre-term birth, and stillbirth) outcomes outside of genetic anomalies [2,4–6]. Although the definition of AMA begins at age 35, the risk of maternal and fetal pregnancy complications increases progressively with age beyond that point, with a drastic increase after age 40. Importantly, despite the mounting evidence and the increased global risk of adverse perinatal outcomes observed, few studies have examined the potential mechanisms underlying this elevated risk in pregnancies of AMA. The work of Wooldridge and colleagues begins to address this gap in the literature [7] and findings from their recent investigation suggest that maternal resistance arteries collected from pregnant women ≥35 years of age were less compliant and had less elastin than arteries obtained from women <35 years of age. Their findings suggest that alterations in maternal resistance vessel stiffness may contribute to increased risk of adverse maternal and fetal outcomes associated with pregnancy at AMA. The authors should be commended for extending prior work in preclinical models to this study of human resistance vessels, which provokes a cascade of questions related to pre-pregnancy vascular function and maternal cardiovascular adaptations to pregnancy in women of AMA.

*Arterial stiffness* describes the rigidity of the arterial wall and the mechanical properties of the arterial vascular system [8]. Arterial stiffening develops from a complex interaction including functional changes in endothelial function and sympathetic nerve activity, and structural remodeling in the arterial wall including changes in collagen and elastin content and wall thickness [9–11]. Importantly, *in vivo* measures of arterial stiffness are the summation of these functional and structural factors, while *in vitro* measures are limited to assessments of the structural components. Of particular interest to the investigation by Wooldridge et al., arterial stiffness assessed *in vivo* and *in vitro* is known to increase with age [12] and is closely associated with cardiovascular disease risk and mortality [13]. Furthermore, increased vascular stiffness assessed *in vivo* in early pregnancy is associated with the development of adverse pregnancy outcomes such as preeclampsia [14,15]. In their *in vitro* approach, Wooldridge et al. [7] obtained omental adipose tissue during scheduled cesarean delivery at term (∼38–39 weeks gestation), isolated omental arteries from this biopsy, and examined collagen and elastin content – the main components of the extracellular matrix within the vascular wall. Previous studies have shown that aging leads to the overproduction of collagen and the degradation of elastin, which contributes to arterial stiffening [16]. Wooldridge et al. also assessed passive mechanical properties (circumferential stress and strain) *in vitro* in isolated omental arteries from pregnant women using pressure myography. Their results showed that elastin content and circumferential strain were lower in AMA compared with pregnant individuals <35 years of age, but collagen content and circumferential stress were not different between groups. However, it should be noted that whether the changes in circumferential stress contributed to differences in the incremental elastic modulus between groups was not assessed. These findings suggest that pregnant women of AMA have increased structural stiffness of resistance arteries at term. Wooldridge et al. postulate that this increased stiffness may contribute to the heightened incidence of adverse maternal and fetal outcomes in pregnancy at AMA. However, whether this reflects an age-associated increase in pre-pregnancy resistance artery stiffness, a maladaptation to the cardiovascular demands of the pregnancy, or a combination of the two, remains unknown.

Although the relation between chronological aging and arterial stiffness is well established, the majority of the data demonstrating this relation focuses on individuals over the age of 60 years and none, to our knowledge, have include pregnant people. Therefore, it is important to highlight the fact that while age ≥35 is considered ‘AMA’, the individuals included in this group had a mean age of 38 years and would be considered ‘young’ in many studies of vascular aging. One of the most novel pieces of the study by Wooldridge and colleagues was their examination of the structural characteristics of resistance (omental) arteries in AMA. Arterial stiffening with aging is not uniform along the arterial tree, due in part to the fact that large arteries such as the aorta have more elastic tissue while resistance arteries have more vascular smooth muscle [17]. Cross-sectional studies illustrate a stronger relation between aortic stiffness and age than that between resistance artery stiffness and age [18,19]. While large artery stiffness is a well-established indicator of age-related cardiovascular events [20], whether this is true for resistance vessels, such as the omental vessels assessed in this study, remains unclear. Therefore, future studies examining the interaction between maternal age and preexisting arterial stiffness are needed.

Alternative to differences in pre-pregnancy stiffness, the authors hypothesize that their findings may reflect differences in cardiovascular adaptations to pregnancy at an AMA. Given the relatively young (compared with aging across the lifespan) and otherwise healthy pre-pregnancy status of the participants included, there is a strong rational to support this hypothesis. Healthy pregnancy requires expansive adaptations in the entire cardiovascular system including decreases in arterial stiffness [21,22] assessed by aortic pulse wave velocity – the gold standard measurement of arterial stiffness in humans – during normal pregnancy [23]. In a previous preclinical study, these same authors reported that circumferential strain of uterine arteries isolated at the end of pregnancy was lower in aged pregnant rats compared with young pregnant rats. However, they found no differences in strain between young non-pregnant and aged non-pregnant rats, mediated by matrix metalloproteinase dysregulation of elastin and collagen and enhanced myogenic vascular tone, leading to reduced vascular compliance [24,25]. These data suggest that decreased elastin content and circumferential strain at term reflects cardiovascular maladaptation to pregnancy in AMA, rather than a baseline effect of aging. Importantly, future studies that include a pre-pregnancy measure and/or longitudinal measures of resistance artery stiffness across pregnancy in healthy women ≥35 years of age are required to fully explore whether the effects observed by Wooldridge et al. are reflective of age-associated pre-pregnancy changes or vascular maladaptation to pregnancy in AMA.

The finding that resistance artery stiffness, assessed *in vitro* at term, is elevated in pregnant individuals ≥35 years of age, presumably as a consequence of cardiovascular maladaptation to pregnancy, initiates a cascade of questions around the mechanisms mediating this outcome. Importantly, this study was not able to discern the role of *in vivo* mechanisms that may have driven the structural changes assessed *in vitro*. Cardiovascular adaptations to pregnancy induce a state of overall reduced total peripheral resistance, mediated in part through enhanced endothelium-dependent vasodilation secondary to increased generation of endothelial-derived vasodilators such as nitric oxide and prostacyclin [26,27] in addition to the structural changes discussed above. Simultaneously, the maternal circulation is exposed to increasing sympathetic nervous system activity [28] and renin–angiotensin system activity [29] which support blood pressure and plasma volume expansion responses to facilitate enhanced blood flow. However, despite an up-regulation of these normally potent vasoconstrictor mechanisms, arterial blood pressure remains relatively stable or slightly decreases due to reductions in baroreflex gain [28] and reduced vascular angiotensin II sensitivity in healthy pregnancy [30]. Impairments in the balance of these vasodilatory and/or vasoconstrictor responses can lead to maternal complications such as gestational hypertension, preeclampsia, and gestational diabetes; however few, if any, human studies have examined normal cardiovascular adaptations to pregnancy in people ≥35 years of age. Increased sympathetic nervous system activity and vasoconstrictor sensitivity both contribute to increases in vessel stiffness outside of pregnancy [31,32], and it is plausible that increases in these mechanisms without appropriate compensatory responses leads to increased resistance vessel stiffness in pregnant individuals of AMA. In support, aged Sprague Dawley rat dams display a greater vasoconstrictive phenotype compared with young dams [25]. Interestingly, a recent meta-regression analysis found that while sympathetic nervous system activity is augmented during normal pregnancy, this was not significantly associated with gestational age [33]. However, whether these normal increases are met with an equally appropriate adaptation, including reduced baroreflex sensitivity and increased endothelium-dependent dilation, to counterbalance the overall stiffening effects in AMA is unknown.

In general, endothelial function decreases with normal aging; however, it is unclear the effect this has during pregnancy in AMA. Similar to studies of arterial stiffening associated with aging, studies of the effects of aging on endothelial function usually define ‘aging’ at midlife (∼55–60 years) and beyond, often after the menopausal transition for women, and women of AMA would still be considered relatively young. Interestingly, aged pregnant rats had enhanced nitric oxide-dependent dilation in mesenteric arteries compared with young pregnant rats at term [25]. Moreover, placental arteries collected from women of AMA at delivery demonstrated increased endothelium-dependent dilation responses to bradykinin compared with young pregnant women [34]. This may indicate that in healthy pregnancies (i.e. without adverse maternal or fetal outcomes) at AMA, the endothelium overcompensates via enhanced nitric oxide production to meet metabolic demand in less compliant vessels. Whether an increased frequency in the inability to upregulate endothelium-dependent dilation in pregnant women ≥35 years underlies the increased incidence of adverse outcomes in these women is entirely unexplored. Although endothelial function was not examined by Wooldridge et al., omental arteries collected from women with a history of preeclampsia – a known state of systemic maternal endothelial dysfunction in pregnancy – found similar irregularities in collagen and elastin compared with vessels from normotensive pregnant women [34]. Thus, the findings from Wooldridge et al. may suggest similar reductions in endothelial function in maternal resistance arteries, contrary to findings seen in murine models. Given the known association of aging with impaired endothelium-dependent dilation, reductions in this mechanism may result in suboptimal vascular adaptations to pregnancy in women of AMA. Future work examining endothelial function of resistance arteries in AMA is required to determine whether alterations in this mechanism contribute to increased vessel stiffness in these women during pregnancy.

**Figure 1:**
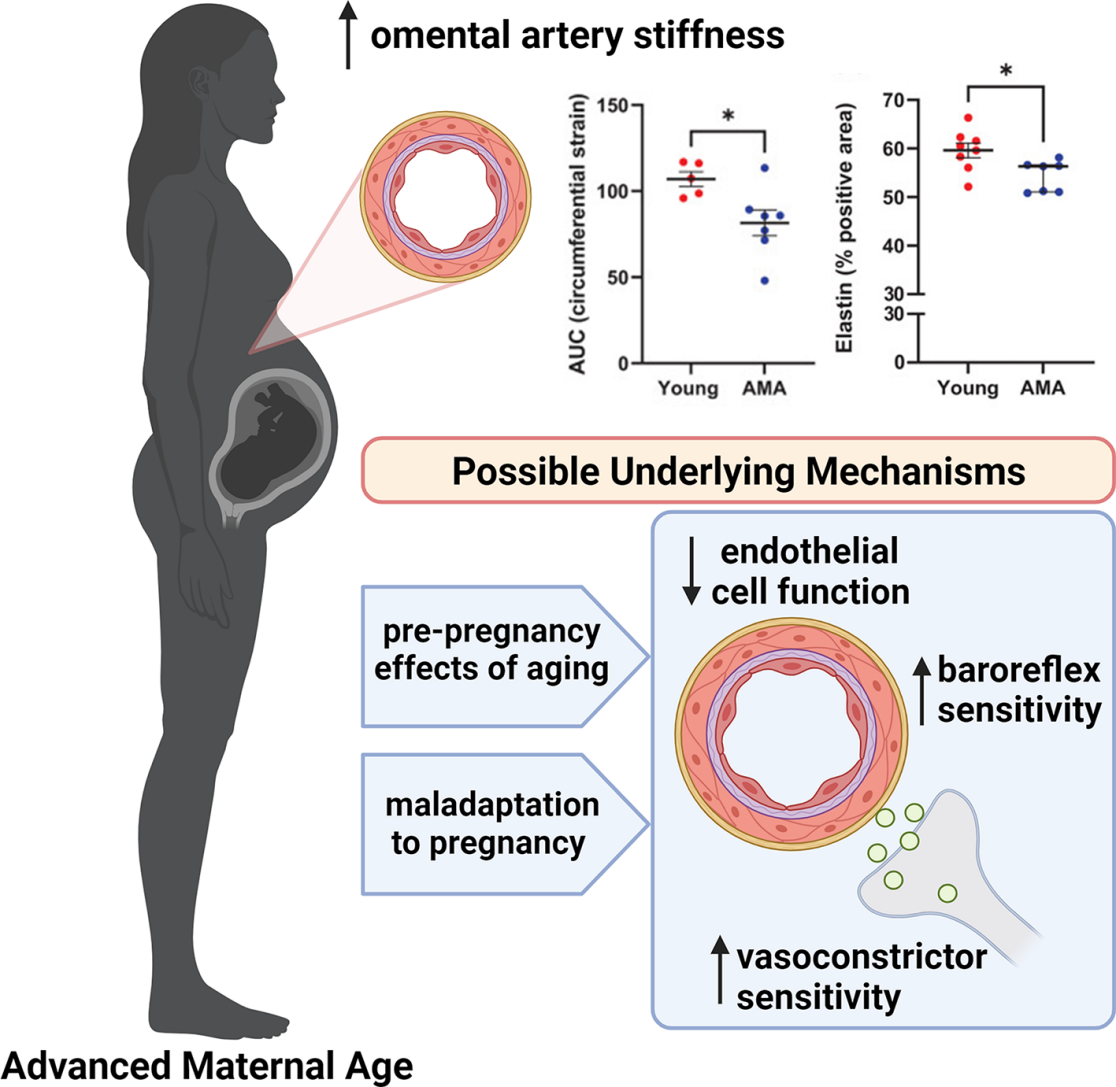
Possible Mechanisms Underlying Increased Resistance Artery Stiffness in Advanced Maternal Age Advanced maternal age (AMA, pregnancy at ≥35 years of age) is associated with increased omental artery stiffness – increased circumferential strain and decreased elastin content – at term (∼38–39 gestational weeks) compared with young (<35 years of age) pregnant women [7]. This increased stiffness may be due to an age-associated increase in pre-pregnancy resistance artery stiffness, a maladaptation to the cardiovascular demands of the pregnancy, or a combination of the two, driving changes in underlying vascular mechanisms. Plausible underlying mechanisms include reduced endothelium-dependent dilation, increased baroreflex sensitivity, and/or increased vasoconstrictor sensitivity, compared with pregnancy in healthy young women.

Overall, the work of Wooldridge and colleagues takes a valuable translational step as it examines omental artery stiffness *in vitro* in human vessels obtained from pregnant women of AMA compared with young pregnant controls. Their findings, that omental arteries from pregnant women ≥35 years of age were less compliant and had less elastin than arteries obtained from women <35 years of age, suggests that alterations in maternal resistance vessel stiffness may contribute to the increased risk of adverse outcomes associated with pregnancy at AMA. These results contribute to our understanding of vascular adaptations to pregnancy in AMA and highlight new directions and novel questions related to the mechanisms underpinning these alterations (Figure 1). It is well established that adverse maternal and fetal outcomes in pregnancy have lasting implications for health across the lifespan. Consequently, as the rates of pregnancy in women of AMA increases, studies of the underlying mechanisms contributing to increased risk of adverse outcomes in these pregnancies are compulsory in order to identify novel approaches to reduce risk and improve lifetime health for these women and their children.

## Abbreviation

AMA: advanced maternal age

## References

[1] Osterman M., Hamilton B., Martin J.A., Driscoll A.K. and Valenzuela C.P. (2021) Births: Final Data for 2020. Natl. Vital Stat. Rep. 70, 1–50 10.15620/cdc:112078 35157571

[2] Laopaiboon M., Lumbiganon P., Intarut N., Mori R., Ganchimeg T., Vogel J.P. et al. (2014) Advanced maternal age and pregnancy outcomes: a multicountry assessment. BJOG: An Int. J. Obstetrics Gynaecol. 121, 49–56 10.1111/1471-0528.12659 24641535

[3] Kim Y.N., Choi D.W., Kim D.S., Park E.C. and Kwon J.Y. (2021) Maternal age and risk of early neonatal mortality: a national cohort study. Sci. Rep. 11, 814 10.1038/s41598-021-80968-4 33436971 PMC7804272

[4] Frick A.P. (2021) Advanced maternal age and adverse pregnancy outcomes. Best Pract. Res. Clin. Obstet. Gynaecol. 70, 92–100 10.1016/j.bpobgyn.2020.07.005 32741623

[5] Jolly M., Sebire N., Harris J., Robinson S. and Regan L. (2000) The risks associated with pregnancy in women aged 35 years or older. Hum. Reprod. 15, 2433–2437 10.1093/humrep/15.11.2433 11056148

[6] Lamminpää R., Vehviläinen-Julkunen K., Gissler M. and Heinonen S. (2012) Preeclampsia complicated by advanced maternal age: a registry-based study on primiparous women in Finland 1997–2008. BMC Pregnancy and Childbirth 12, 47 10.1186/1471-2393-12-47 22687260 PMC3495042

[7] Wooldridge A.L., Chan C., Spaans F., Quon A., Steinback C.D., Davenport M.H. et al. (2023) Increased stiffness of omental arteries from late pregnant women at advanced maternal age. Biosci. Rep. 43, 10.1042/BSR20230819 37493195 PMC10447229

[8] Avolio A. (2013) Arterial stiffness. Pulse 1, 14–28 10.1159/000348620 26587425 PMC4315342

[9] Duprez D.A. (2010) Arterial stiffness and endothelial function: key players in vascular health. Hypertension 55, 612–613 10.1161/HYPERTENSIONAHA.109.144725 20083729

[10] Okada Y., Galbreath M.M., Shibata S., Jarvis S.S., VanGundy T.B., Meier R.L. et al. (2012) Relationship between sympathetic baroreflex sensitivity and arterial stiffness in elderly men and women. Hypertension 59, 98–104 10.1161/HYPERTENSIONAHA.111.176560 22106403 PMC3241909

[11] Zieman S.J., Melenovsky V. and Kass D.A. (2005) Mechanisms, pathophysiology, and therapy of arterial stiffness. Arterioscler. Thromb. Vasc. Biol. 25, 932–943 10.1161/01.ATV.0000160548.78317.29 15731494

[12] McVeigh G.E., Bratteli C.W., Morgan D.J., Alinder C.M., Glasser S.P., Finkelstein S.M. et al. (1999) Age-related abnormalities in arterial compliance identified by pressure pulse contour analysis: aging and arterial compliance. Hypertension 33, 1392–1398 10.1161/01.HYP.33.6.1392 10373222

[13] Laurent S., Boutouyrie P., Asmar R., Gautier I., Laloux B., Guize L. et al. (2001) Aortic stiffness is an independent predictor of all-cause and cardiovascular mortality in hypertensive patients. Hypertension 37, 1236–1241 10.1161/01.HYP.37.5.1236 11358934

[14] Nuckols V.R., Holwerda S.W., Luehrs R.E., DuBose L.E., Stroud A.K., Brandt D. et al. (2020) Beat-to-beat blood pressure variability in the first trimester is associated with the development of preeclampsia in a prospective cohort: relation with aortic stiffness. Hypertension 76, 1800–1807 10.1161/HYPERTENSIONAHA.120.15019 32951467 PMC7706825

[15] Phan K., Gomez Y.H., Gorgui J., El-Messidi A., Gagnon R., Abenhaim H.A. et al. (2023) Arterial stiffness for the early prediction of pre-eclampsia compared with blood pressure, uterine artery Doppler and angiogenic biomarkers: a prospective cohort study. BJOG: An Int. J. Obstetrics Gynaecol. 130, 932–940 10.1111/1471-0528.17430 36807704

[16] Lakatta E.G. (2003) Arterial and cardiac aging: major shareholders in cardiovascular disease enterprises: Part III: cellular and molecular clues to heart and arterial aging. Circulation 107, 490–497 10.1161/01.CIR.0000048894.99865.02 12551876

[17] Tucker W.D., Arora Y. and Mahajan K. (2023) Anatomy, blood vessels. Available from: https://www.ncbi.nlm.nih.gov/books/NBK470401/29262226

[18] Fortier C., Mac-Way F., Desmeules S., Marquis K., De Serres S.A., Lebel M. et al. (2015) Aortic-brachial stiffness mismatch and mortality in dialysis population. Hypertension 65, 378–384 10.1161/HYPERTENSIONAHA.114.04587 25452473

[19] Kimoto E., Shoji T., Shinohara K., Inaba M., Okuno Y., Miki T. et al. (2003) Preferential stiffening of central over peripheral arteries in type 2 diabetes. Diabetes 52, 448–452 10.2337/diabetes.52.2.448 12540620

[20] Ben-Shlomo Y., Spears M., Boustred C., May M., Anderson S.G., Benjamin E.J. et al. (2014) Aortic pulse wave velocity improves cardiovascular event prediction: an individual participant meta-analysis of prospective observational data from 17,635 subjects. J. Am. Coll. Cardiol. 63, 636–646 10.1016/j.jacc.2013.09.063 24239664 PMC4401072

[21] Mashini I.S., Albazzaz S.J., Fadel H.E., Abdulla A.M., Hadi H.A., Harp R. et al. (1987) Serial noninvasive evaluation of cardiovascular hemodynamics during pregnancy. Am. J. Obstet. Gynecol. 156, 1208–1213 10.1016/0002-9378(87)90146-3 3578440

[22] Robson S.C., Hunter S., Boys R.J. and Dunlop W. (1989) Serial study of factors influencing changes in cardiac output during human pregnancy. Am. J. Physiol.-Heart Circulatory Physiol. 256, H1060–H1065 10.1152/ajpheart.1989.256.4.H1060 2705548

[23] Mahendru A.A., Everett T.R., Wilkinson I.B., Lees C.C. and McEniery C.M. (2014) A longitudinal study of maternal cardiovascular function from preconception to the postpartum period. J. Hypertens. 32, 849–856 10.1097/HJH.0000000000000090 24406777

[24] Wooldridge A.L., Pasha M., Chitrakar P., Kirschenman R., Quon A., Spaans F. et al. (2022) Advanced maternal age impairs uterine artery adaptations to pregnancy in rats. Int. J. Mol. Sci. 23, 9191 10.3390/ijms23169191 36012456 PMC9409016

[25] Care A.S., Bourque S.L., Morton J.S., Hjartarson E.P. and Davidge S.T. (2015) Effect of advanced maternal age on pregnancy outcomes and vascular function in the rat. Hypertension 65, 1324–1330 10.1161/HYPERTENSIONAHA.115.05167 25916720

[26] Morton J.S. and Davidge S.T. (2013) Arterial endothelium-derived hyperpolarization: potential role in pregnancy adaptations and complications. J. Cardiovasc. Pharmacol. 61, 197–203 10.1097/FJC.0b013e31827b6367 23188131

[27] Valdes G., Kaufmann P., Corthorn J., Erices R., Brosnihan K.B. and Joyner-Grantham J. (2009) Vasodilator factors in the systemic and local adaptations to pregnancy. Reprod. Biol. Endocrinol. 7, 79 10.1186/1477-7827-7-79 19646248 PMC2739214

[28] Usselman C.W., Skow R.J., Matenchuk B.A., Chari R.S., Julian C.G., Stickland M.K. et al. (1985) Sympathetic baroreflex gain in normotensive pregnant women. J. Appl. Physiol. 119, 468–474, 2015 10.1152/japplphysiol.00131.2015 PMC4556837 26139215

[29] Brooks V.L., Fu Q., Shi Z. and Heesch C.M. (2020) Adaptations in autonomic nervous system regulation in normal and hypertensive pregnancy. Handb. Clin. Neurol. 171, 57–84 10.1016/B978-0-444-64239-4.00003-5 32736759 PMC9261029

[30] Irani R.A. and Xia Y. (2011) Renin angiotensin signaling in normal pregnancy and preeclampsia. Semin. Nephrol. 31, 47–58 10.1016/j.semnephrol.2010.10.005 21266264 PMC3275085

[31] Holwerda S.W., Luehrs R.E., DuBose L., Collins M.T., Wooldridge N.A., Stroud A.K. et al. (2019) Elevated muscle sympathetic nerve activity contributes to central artery stiffness in young and middle-age/older adults. Hypertension 73, 1025–1035 10.1161/HYPERTENSIONAHA.118.12462 30905199 PMC6937199

[32] Schiffrin E.L. (2020) How Structure, mechanics, and function of the vasculature contribute to blood pressure elevation in hypertension. Can. J. Cardiol. 36, 648–658 10.1016/j.cjca.2020.02.003 32389338

[33] Greenwall K.M., Brislane A., Matenchuk B.A., Sivak A., Davenport M.H. and Steinback C.D. (2023) Muscle sympathetic nerve activity during pregnancy: a systematic review and meta-analysis. Physiol. Rep. 11, e15626 10.14814/phy2.15626 36905144 PMC10006587

[34] Suzuki Y., Yamamoto T., Mabuchi Y., Tada T., Suzumori K., Soji T. et al. (2003) Ultrastructural changes in omental resistance artery in women with preeclampsia. Am. J. Obstet. Gynecol. 189, 216–221 10.1067/mob.2003.445 12861165

